# Analyzing Homogeneity of Highly Viscous Polymer Suspensions in Change Can Mixers

**DOI:** 10.3390/polym16182675

**Published:** 2024-09-23

**Authors:** Michael Roland Larsen, Erik Tomas Holmen Olofsson, Jon Spangenberg

**Affiliations:** 1 Department of Civil and Mechanical Engineering, Technical University of Denmark, 2800 Kongens Lyngby, Denmark; ethol@dtu.dk (E.T.H.O.); josp@dtu.dk (J.S.); 2 Dansac A/S, 3480 Fredensborg, Denmark; 3 Haldor Topsoe A/S, 2800 Kongens Lyngby, Denmark

**Keywords:** mixing, suspensions, homogeneity, computational fluid dynamics, parameter investigation

## Abstract

The mixing of highly viscous non-Newtonian suspensions is a critical process in various industrial applications. This computational fluid dynamics (CFD) study presents an in-depth analysis of non-isothermal mixing performance in change can mixers. The aim of the study was to identify parameters that significantly influence both distributive and dispersive mixing in these mixers, which are essential for optimizing industrial mixing processes. The study employed a numerical design of experiments (DOE) approach to identify the parameters that most significantly influence both distributive and dispersive mixing, as measured by the Kramer mixing index ($M_{Kramer}$) and the Ica Manas-Zloczower mixing index $\left(\bar{\lambda_{MZ}}\right)$. The investigated parameters included mixing time, number of arms, arm size ratio, revolutions per minute (RPM), z-axis rotation, z-axis movement, and initial and mixing temperatures. The methodology involved employing the bootstrap forest algorithm for predicting the mixing indices, achieving an $R^{2}$ of 0.949 for $M_{Kramer}$ and an $R^{2}$ of 0.836 for $\bar{\lambda_{MZ}}$. The results indicate that the z-axis rotation has the greatest impact on both distributive and dispersive mixing. An increased number of arms negatively impacted $\lambda_{MZ}$, but had a small positive effect on $M_{Kramer}$. Surprisingly, in this study, neither the initial temperature of the material nor the mixing temperature significantly impacted the mixing performance. These findings highlight the relative importance of operational parameters over traditional temperature factors and provide a new perspective on mixing science.

## 1. Introduction

Mixing fluids and powders is a fundamental process that has been present for centuries in various industries. Achieving uniformity in such mixtures, also known as homogeneity, is critical to ensure consistent quality and performance in food, concrete, medical device, and catalyst industries.

Homogeneity in particle-based slurries can be quantified using a distributive and dispersive mixing index [1]. Distributive mixing refers to the uniform component distribution within the matrix, ensuring a consistent spatial arrangement. In parallel, dispersive mixing aims to intentionally reduce the size of cohesive components, particularly clusters of solid particles, to enhance homogeneity.

Mixing indices have been developed to quantitatively evaluate the level of mixing in fluid-powder mixtures. Among them, the distributive mixing index is one of the earliest, originating with Lacey in the mid-19th century, who proposed using standard deviation from a concentration to estimate mixing quality [2,3]. This idea was later expanded upon by Kramer [4] and Ashton-Valentin [5]. Despite originating in a different era, the Kramer mixing index remains a vital tool in contemporary research; it has, e.g., recently been used to in cooperation with computational fluid dynamics (CFD) [6]. With the advent of CFD, newer approaches, such as the cluster distribution index, have been developed to evaluate the distribution of Lagrangian particles in a mixer [7,8]. Another particle-based approach is the “Scale of Segregation” developed by P.V. Danckwerts [9] and used in CFD simulations by Connelly and Kokini in 2007 [10].

The dispersive mixing index quantifies a mixer’s ability to break up agglomerates, which is a critical factor in ensuring product quality. The efficiency of agglomerate breakage for highly viscous fluids depends on the type of flow, with elongation flow being the most effective [11]. In 1992, Ica Manas-Zloczower introduced the widely used mixing index $\lambda_{MZ}$, which describes the relationship between simple vorticity flow ${(\lambda }_{MZ}\approx 0)$ and simple elongation flow $\left(\lambda_{MZ}=1\right)$ [12]. This mixing index has been extensively used in the literature [13,14].

Viscosity reduction via heat is a well-established phenomenon in the mixing literature [15,16]. This phenomenon is important for increasing the Reynolds number and, thereby, the distributive mixing efficiency [17]. Fluid friction, also known as viscous dissipation, tends to be neglected despite being a notable factor when dealing with highly viscous fluids [17]. The impact of viscous dissipation should, therefore, not be ignored, as demonstrated in past studies [15,18].

In the context of evaluating numerous parameters, machine learning techniques can be highly effective. Bootstrap Forest, a commercialized adaptation based on the random forest algorithm, excels in evaluating multiple parameters and their interactions. For instance, Duan and Takemi [19] demonstrated its utility in predicting urban surface roughness aerodynamic parameters, showing its robustness in handling complex, nonlinear relationships. Similarly, Ganesh et al. [20] applied random forest regression to accurately estimate fluid flow characteristics in curved pipes, highlighting its capability to reduce computational costs while maintaining high accuracy in predictions. Both studies utilized the random forest algorithm to achieve significant insights.

This study aims to expand the knowledge of dispersive and distributive mixing of highly viscous non-Newtonian suspensions in “change can mixers”. In this regard, a CFD model was developed and exploited to perform a numerical design of experiments (DOE). The evaluation of the homogeneity of the suspension was performed by Kramer’s and Ica Manas-Zloczower’s mixing indexes, and seven process parameters were included in the DOE that the Taguchi and definitive screening design techniques inspired. The results of the DOE were analyzed by a bootstrap forest algorithm to evaluate the significance of each process parameter, leading to the development of a predictive model for the mixing indices. Following this introduction, the paper is organized as follows: Section 2 elucidates the methodology setup, encompassing comprehensive information about the numerical model and DOE. Section 3 presents and analyzes the study’s findings, emphasizing the influence of the investigated parameters on the mixing performance. Ultimately, Section 4 summarizes the conclusions drawn from the study, underscoring the implications and significance of the findings.

## 2. Methodology

### 2.1. Material and Mixer Information

The suspension that the CFD model simulated has been characterized experimentally in terms of density, heat capacity and conductivity, as well as rheology. The latter is presented in the next subsection. The suspension was a combination of a highly viscous polymer fluid and a powder blend consisting of both colloidal and microscale particles. The specific fluid and powder are protected intellectual property and can, therefore, not be disclosed. In this regard, it is important to note that the primary focus is on the mixing process dynamics of the highly viscous polymer suspension rather than on a detailed analysis of the material composition itself. Table 1 shows the physical data of the mixed material. The density was measured with a “Sartorius YDK03 Density Kit for Analytical Balances” using the Archimedes principle. The heat capacity and conductivity were measured on a “TCi-3-A” from C-Therm Technologies Ltd. (Fredericton, NB, Canada) using the modified transient plane source (MTPS) method.

**Figure 1:**
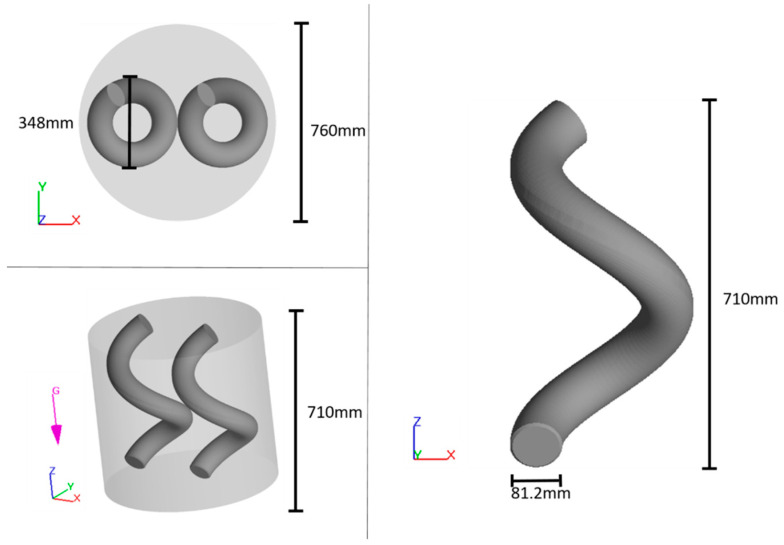
Illustration of the change can mixer (**left**) and mixing arm (**right**).

Details about the change can mixer that CFD model simulates are illustrated in Figure 1. Heat is introduced into the system from the wall and bottom of the mixer. The top does not apply heat due to the physical setup where the rotor arms are mounted. The change can mixer operates under vacuum conditions to prevent air from becoming trapped in the mixing material. During operation, the mixer’s top lid rotates clockwise and spins around the z-axis fulcrum, while the arms themselves also rotate around their own z-axes. For clarity throughout this study, the top lid’s z-axis revolutions per minute is denoted as RPM and the rotation around the arm’s z-axis per minute is referred to as the z-axis rotation.

### 2.2. Numerical Model

The CFD model simulates the mixing of a suspension in an industrial-scale mixer using the commercial software, FLOW-3D (2022R1).

**Figure 2:**
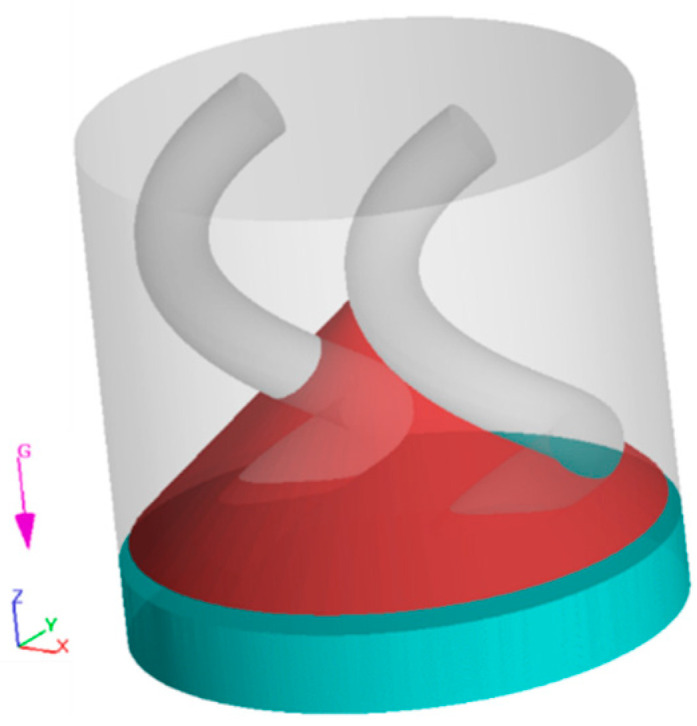
CFD model at t=0. The blue/turquoise mass represents the fluid, while the red mass represents the powder.

This software, known for its accurate fluid dynamics simulations, was chosen for its advanced TruVOF technique, which is particularly effective in modeling the free surface flows that are applied for these simulations. The software has previously been used to simulate other processes, such as 3D printing [21] and concrete casting [22]. Figure 2 shows an example of the simulation setup at t=0. The red domain represents the powder component, while the blue/turquoise part represents the suspending fluid. The rotation begins at t>0 and remains constant throughout the simulation, and a no-slip boundary condition is applied on all solid surfaces, including the rotational arms. The computational domain is meshed with a uniform grid consisting of ~134,000 cells. The number of cells was determined through a mesh sensitivity analysis. To ensure the reliability of our simulations, all models achieved satisfactory residual and convergence levels, indicating that the results can be trusted for higher accuracy.

The simulation is computed as a transient non-isothermal flow because the viscosity is temperature-dependent. The material model substance is considered incompressible, hence, the density ρ is assumed constant. Thus, the flow is computed by considering mass, momentum, and energy conservation, as shown below in (1)–(4).

(1) ∇·v=0

(2) $$\rho \frac{Dv}{Dt}=-\nabla p-\left[\nabla \cdot \tau \right]+\rho G$$

(3) $$\rho C_{p}\frac{DT}{Dt}=-\left(\nabla \cdot q\right)-(\tau :\nabla v)$$

(4) q=−k∇T

where the pressure, velocity vector, and gravitational vector are denoted as p, v, and **G**, respectively. The gravitational force is defined as $\left(0,0,-9.82\right)\frac{m}{s^{2}}$. The heat flux is represented as q, the specific heat capacity by $C_{p}$ and k is the thermal conductivity. Additionally, the deviatoric stress tensor τ, is calculated as seen in (5) and (6).

(5) $$\tau =2\mu \left(\dot{\gamma },T\right)D$$

(6) $$D=\frac{1}{2}\left(\nabla v\right)+\nabla v^{T}$$

where D is the deformation rate tensor and $\dot{\gamma }$ is the shear rate calculated from the trace of D, $\dot{\gamma }=\sqrt{2tr(D^{2})}$. To compute the equations from above, the software uses the finite volume method to discretize the governing equations. The free surface is calculated using the volume of fluid (VOF) technique [23]. The viscous stress and pressure are both solved implicitly, while the equation of advection is solved explicitly with 2nd-order accuracy.

The material was simulated with the properties described in the previous section (excl. rheology). The simulated material exhibits a viscoplastic behavior and is modeled using the Herschel–Bulkley model, with a slight modification to account for temperature dependency. Equation (7) describes the modified Herschel–Bulkley viscosity model, where $\tau_{0}$ is the yield stress, $k_{HB}$ is the consistency index, n is the flow index, and $\mu_{0}$ as the initial viscosity. The temperature-dependent energy function, E in Equation (8) is represented by the empirically adjusted constants a and c. The reference temperature and fluid temperature are denoted by $T^{ref}$ and T, respectively. In this context, the term “reference temperature” ($T^{ref}$) is an empirical fitting factor utilized within the Flow3D model.

(7) $$\left(\dot{\gamma },T\right)=\left\{\begin{array}{c} \mu_{max}\;\begin{array}{ccc} , & \;for & \dot{\gamma }\leq {\dot{\gamma }}_{c} \end{array}\\ \begin{array}{ccc} \mu_{0}E{\left(T\right)}^{n}{\dot{\gamma }}^{1-n}+\frac{\tau_{0}}{\dot{\gamma }}, & for & \dot{\gamma }>{\dot{\gamma }}_{c} \end{array} \end{array}\right.$$

(8) $$E\left(T\right)=\exp\left(a\left(\frac{T^{ref}}{T}-c\right)\right)$$

**Figure 3:**
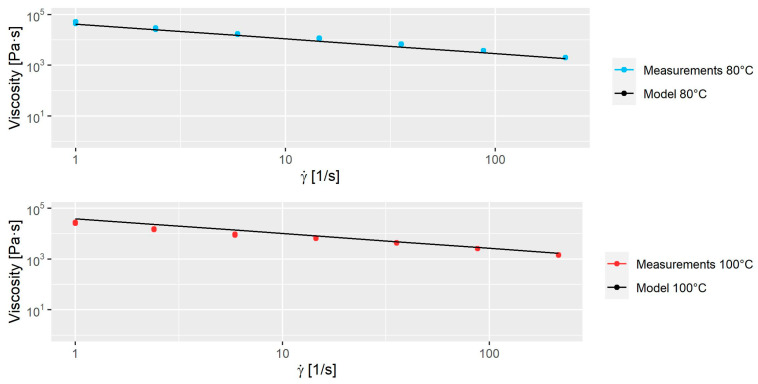
The model plotted to measurements at 80 and 100 °C.

The applied values used for this model are shown in Table 2. The model was fitted to rheological measurements at 80 °C and 100 °C with shear rates between $1\;s^{-1}$ and $215.7\;s^{-1}$. The rheological experiments were performed on a Dynisco LCR-7001 capillary rheometer with a 1 mm × 20 mm capillary unit. In Figure 3, the comparison between experimental data and model is seen. The viscosity of a suspension changes depending on the powder volume fraction, as demonstrated by Einstein in 1906 [24]. While this interaction affects the mixing (especially in the initial stages), this study will assume that the interaction has a negligible effect on the numerical results and is therefore not considered.

The evaluation of the mixing was done using both a dispersive and distributive mixing indices. The Kramer index [4] was employed to measure the distributive mixing. The index requires an artificial dimensionless scalar concentration, $c_{i}$, with zero diffusion to ensure that the only factor propelling the mixing is the rotation itself. The powder and fluid were assigned a concentration of 1 and 0, respectively. This concentration did not affect the physical properties such as viscosity and density. The mean of the concentration $\bar{c}\;$, was 0.5. The dimensionless concentration $\hat{c_{i}}$ was calculated using Equation (9) from the scalar concentration. The dimensionless variance and $M_{Kramer}$ can be found through Equations (9)–(11):

(9) $$\hat{c_{i}}=\frac{c_{i}-\bar{c}}{\bar{c}}$$

(10) $$S^{2}=\frac{1}{\left(N_{f}-1\right)}\sum_{i=1}^{N_{f}}{\left(\hat{c_{i}}\right)}^{2}$$

(11) $$M_{Kramer}=\frac{\sigma_{0}-S}{\sigma_{0}-\sigma_{r}\;}$$

$S^{2}$ is the dimensionless variance, and $N_{f}$ represents the number of elements that contain fluid. The $\sigma_{0}$ and $\sigma_{r}$ are defined as $\sigma_{0}{=\left(P\cdot \left(1-P\right)\right)}^{\frac{1}{2}}$ and $\sigma_{r}=\frac{\sigma_{0}}{N_{f}}$, respectively, where P is the average concentration of the powder, which is 0.5. A large $M_{Kramer}$ value indicates a homogeneous mixture.

The evaluation of the dispersive mixing was performed with the Manas–Zlacower mixing index $\lambda_{MZ}$ shown in Equation (12). It was calculated by the shear rate and the vorticity, ω. The index indicates which type of flow is currently present. It is desirable to get as many elements as close to 1 as possible as it is proven that elongation flow breaks up agglomerates faster [25,26].

(12) $$\lambda_{MZ}=\frac{\left|\dot{\gamma }\right|}{\left|\dot{\gamma }\right|+|\omega |}$$

### 2.3. Design of Experiments

DOE is a systematic method used to investigate process output by varying multiple parameters. This study used a definitive screening design to cover pre-set parameters and minimize simulation time. This choice was driven by the unique ability of Definitive Screening Design to provide a comprehensive yet efficient exploration of the process space, effectively assessing main effects and factor interactions. Furthermore, it is suitable for handling nonlinear effects [27]. Previous studies using DOE yielded satisfactory results [28,29]. The definitive screening design initially suggested 18 simulations. Some of the simulations were very computationally heavy, so while waiting for these simulations to finish, an additional 17 simulations were executed in order to cover even more of the parameter space. This brings the total number of simulations to 35, each identified by a unique simulation number (Sim. No.). The data treatment of the DOE was performed in SAS JMP^®^, employing the standard least squares method. The process parameters and their variation are presented below:

The initial and mixing temperature is known to reduce the viscosity of fluids [17,30], which will increase Reynolds number and potentially improve mass transfer during mixing [17]. The initial temperature is modified only for the suspending fluid, as the powder requires a constant initial temperature of 33 °C. The initial temperature of the fluid and the mixing temperature are varied between 20 °C and 80 °C. The RPM value and the z-axis rotation also affect the suspension mixing, as demonstrated by [31]. In this study, the RPM value was varied between 3 and 30, while the z-axis rotation was altered between 0 and 157.5. This study also explored the influence of moving the rotational arm 10 cm up and down along the z-axis. This z-axis movement had a frequency that was varied between 0 and 6 per minute. Finally, the number of arms in the mixer were varied from 1 to 3, and the size ratio was varied between 2/3 and 1.1. The size ratio represents that one arm in the mixer has a diameter that is given by the size ratio multiplied by the original diameter of 81.2 mm, cf. Figure 1. In Table 3, the numerical DOE is shown. Note that the mixing time is 300 s in all scenarios.

### 2.4. Bootstrap Forest

A predictive model, specifically designed for distributive and dispersive mixing indices, was developed using the bootstrap forest algorithm in the JMP 16 Pro software, a type of random forest algorithm [32]. The algorithm is an ensemble learning method that combines multiple decision trees, $F_{i}(x)$, to create a more accurate and robust model. The bootstrap forest algorithm employs bootstrapping, a resampling technique that creates multiple subsets of the original dataset by repeatedly sampling with replacement. Each subset is used to train an individual decision tree within the forest, enhancing the model’s robustness and accuracy. The predicted value, $\overset{ˇ}{y}$, is obtained by averaging the predictions from all the individual trees in the forest, as seen in Equation (13).

(13) $$\overset{ˇ}{y}=\frac{1}{N}\sum_{i=1}^{N}F_{i}(x)$$

where N is the number of trees, and $F_{i}(x)$ is the prediction of the ith tree for the input vector row x that specifies the specific parameter found in Table 3.

The selected data to be evaluated by the algorithm was a balanced blend of systematic and random sampling. Specific time steps were selected at regular intervals and supplemented by additional points randomly selected via a random number generator. Each simulation yielded 26 data points, 11 chosen systematically and 15 chosen randomly. This method ensured computational efficiency and controlled memory usage, which was particularly important given the intensive computational requirements and memory capacity necessary for processing the 35 simulations. Note that the treatment of time-dependency in the analysis is described in Section 3.5.1 and Section 3.5.2 for dispersive and distributive mixing, respectively.

The collected data was then partitioned into training and validation subsets, following a conventional 70-30 split in line with standard machine learning practice [33]. The coefficient of determination, $R^{2}$, was calculated to assess the performance of the fit of the predictive model:

(14) $$R^{2}=1-\frac{\sum_{i=1}^{N}{\left(y_{i}-{\overset{ˇ}{y}}_{i}\right)}^{2}}{\sum_{i=1}^{N}{\left(y_{i}-\bar{y}\right)}^{2}}$$

where $y_{i}$ denotes a specific simulation observation and $\bar{y}$ represents the mean value of all observations for the output function; this calculation serves as a pivotal metric for assessing the adequacy of the predictive model in replicating the observed data.

Before splitting the data into training and validation, a predictor screening was carried out, which essentially is a bootstrap forest analysis without validation. This screening quantified each parameter’s individual contribution. A parameter contribution threshold of 5% was set. Parameters with an average contribution below this threshold during the predictor screening were excluded, while those that made over a 5% contribution were included in the full bootstrap forest analysis. This strategy helped reduce the model’s complexity and improved interpretability by eliminating less impactful variables. The 5% threshold was established based on the same criteria used in linear regression, where parameters with a *p*-value below 0.05 are excluded.

## 3. Results and Discussion

This section delineates the findings of the analysis concerning the mixing performance in change can mixers. Specifically, the velocity profile, temperature profile, dispersive mixing index, and distributive mixing index will be delved into. Given the intricacy of presenting 35 distinct simulations, the focus will be on two representative simulations for illustration purposes: Sim. No. 7 and Sim. No. 35. These two simulation were chosen as they are quite different.

Subsequently, the parameter study with all 35 simulations utilizing the bootstrap forest analysis is presented. Initially, the influence of parameters on the dispersive mixing index is scrutinized. Next, the impact of parameters on the distributive mixing index is outlined.

### 3.1. Velocity Profile

**Figure 4:**
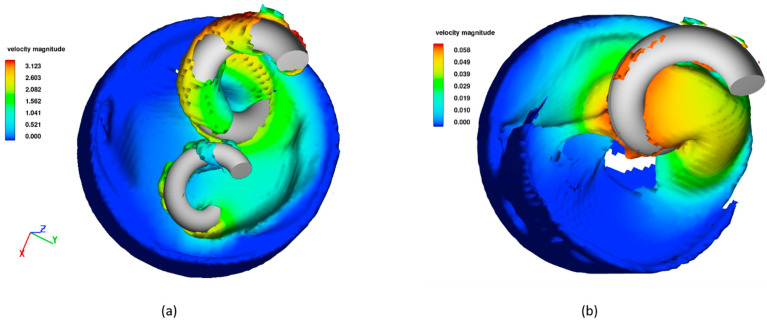
Velocity profiles at 60 s for (**a**) Sim. No. 7 and (**b**) Sim. No. 35. The velocity values are in m/s.

The velocity profiles for two representative simulations, Sim. No. 7 and 35, are depicted in Figure 4 at t=60 s. The maximum velocity magnitude and velocity gradients are substantially lower in the simulation with one arm as compared to the simulation with two arms. This outcome was anticipated due to the lower RPM and z-axis rotation values utilized in Sim. No. 35. Both simulations illustrate that most flow takes place near the mixing arms, which is also expected. In addition, Sim. No. 7 displays the highest velocities around the large arm, which is due to the arms having the same angular velocity, resulting in a higher velocity for the large arm at the outer edge.

### 3.2. Temperature Profile

**Figure 5:**
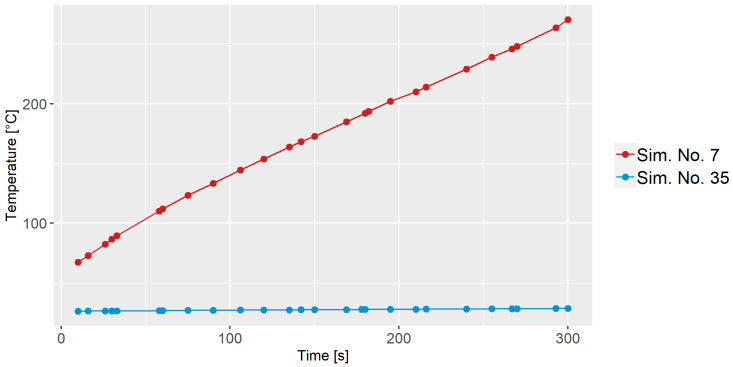
Average fluid temperature for Sim. No. 7 and Sim. No. 35 as a function of time.

The temperature profile analysis compares the average fluid temperature for Sim. No. 7 and 35, as illustrated in Figure 5. Both simulations commence with different initial heat values and wall temperatures. Sim. No. 7 starts with an initial temperature of 80 °C, identical to the wall temperature, while Sim. No. 35 begins with an initial temperature of 20 °C, which also aligns with the wall temperature. In Sim. No. 7, the temperature surpasses the wall temperature, which is above 250 °C. This can be ascribed to viscous dissipation, a phenomenon where heat is generated due to fluid friction during the mixing process. It is important to note that temperatures reaching 250 °C or higher could potentially ruin the material being mixed. Such elevated temperatures may lead to degradation or other undesirable changes in the material properties. Conversely, in Sim. No. 35, the lower RPM and z-axis rotation values lead to minimal or no viscous dissipation. As a result, the temperature remains below 30 °C.

### 3.3. Dispersive Mixing

**Figure 6:**
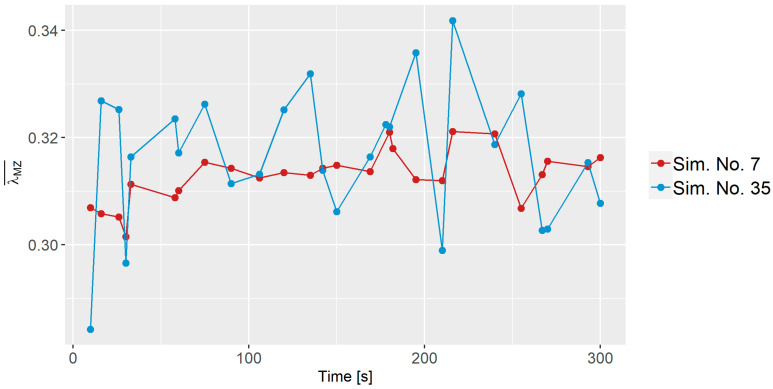
Dispersive mixing index for Sim. No. 7 and 35 as a function of time.

Figure 6 presents the average dispersive mixing index $\bar{\lambda_{MZ}}$ for Sim. No. 7 and 35 at various time intervals. The dispersive mixing index values for Sim. No. 7 primarily ranges between 0.30 and 0.32, while for Sim. No. 35, they approximately span from 0.28 to 0.34. It is interesting to note that the mean value of the dispersive index is fairly similar for Sim. No. 7 and 35, even though they have quite different process parameters. However, that is not the case for all simulations, e.g., the mean value of Sim. No. 23 is 0.26.

### 3.4. Distributive Mixing

**Figure 7:**
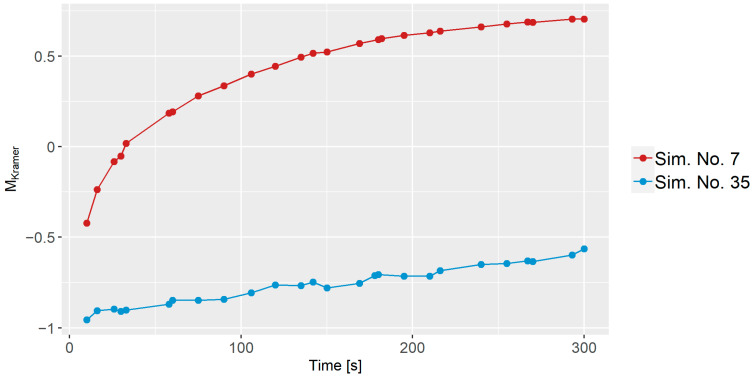
Distributive mixing index for Sim. No. 7 and Sim. No. 35 compared to time.

In Figure 7, the $M_{Kramer}$ values (i.e., the distributive mixing index) as a function of time is shown for Sim. No. 7 and 35. Both start below 0 due to the initial high standard deviation and subsequently, both simulations exhibit an increase in the $M_{Kramer}$ values, which indicates that the mixes become more homogeneous. Sim. No. 7 undergoes a significantly faster mixing process compared to Sim. No. 35. After 300 s, Sim. No. 7 reaches around 0.75, while Sim. No. 35 only attains approximately −0.5. This observation aligns with the velocity profile analysis, where Sim. No. 7 exhibits a faster velocity compared to Sim. No. 35, and thus faster mass transfer. The results highlight that the mixing process is time-dependent but that other factors, such as the specific parameters, also play a crucial role in influencing the mixing behavior.

### 3.5. Bootstrap Forrest

#### 3.5.1. Dispersive Modeling

Since time primarily introduced fluctuations rather than significant alterations in the modeling of the dispersive mixing index, as seen in Figure 6, it was excluded from this analysis. Nevertheless, when analyzing the dispersive mixing index, all 26 lambda values for each simulation were considered instead of using an average to better understand the estimated capabilities of the predictive model. The process parameters from Table 3 were considered in the analysis. An initial investigation was conducted using the predictor screening analysis to minimize the number of parameters. Parameters with a contribution of less than 5% were not selected for further analysis.

**Figure 8:**
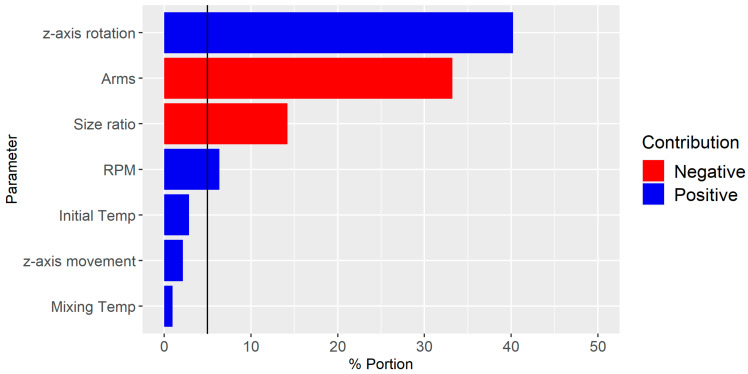
Predictor screening analysis of the influence of each parameter on dispersive mixing. Note the significance line at 5%.

Figure 8 illustrates a bar chart of the predictor screening analysis, highlighting the influence of each parameter on dispersive mixing. It is evident that the z-axis rotation has the highest impact, followed by the number of arms, Size ratio, and RPM, all of which exceed the significance line. Conversely, z-axis movement, initial temperature, and mixing temperature did not exhibit significant contributions and were not considered further.

**Figure 9:**
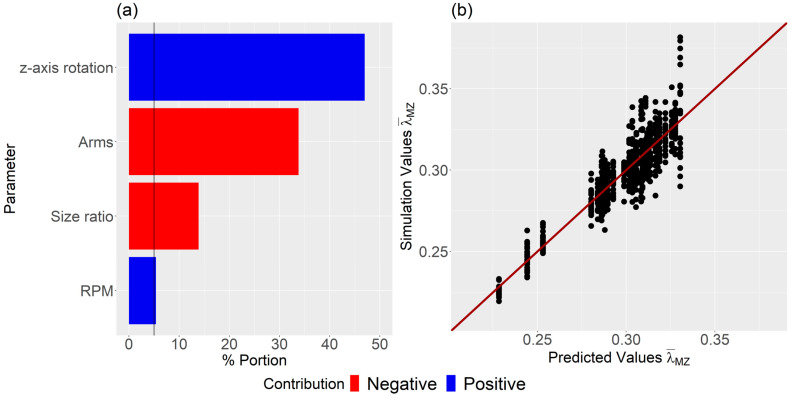
(**a**) Influence of each parameter on dispersive mixing, and (**b**) comparison between predicted and simulation values. Note the significance line at 5% in (**a**).

Subsequently, the bootstrap forest algorithm was executed, focusing only on the four significant parameters, cf. Figure 9a. This second analysis aimed to evaluate these four parameters’ influence and generate the predictive model. The impact sequence of the individual parameters on dispersive mixing remained unchanged, indicating that the threshold value was thoughtfully established. The $R^{2}$ value for the validation set was found to be 0.836. Figure 9b presents a comparison of predicted values and simulation outcomes, demonstrating commendable accuracy.

**Figure 10:**
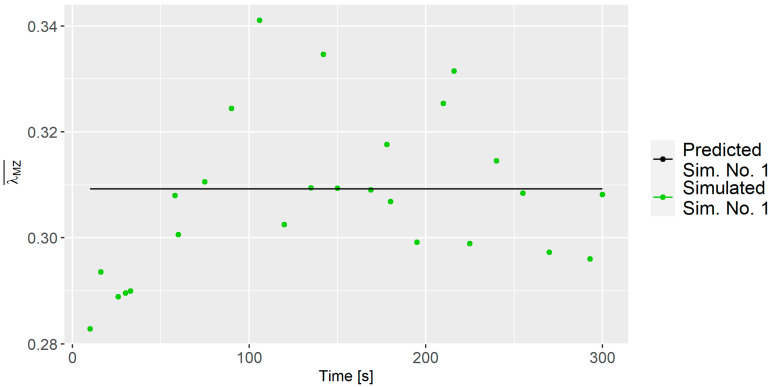
$\bar{\lambda_{MZ}}$ values for Batch 12 with predicted and simulation values.

**Figure 11:**
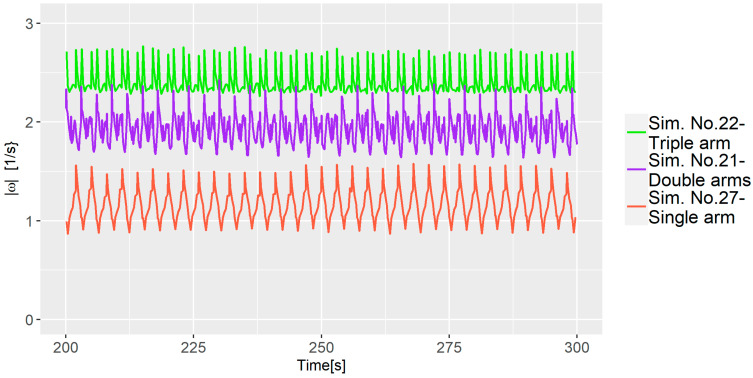
Vorticity comparison for three simulations with varying numbers of arms.

It is important to mention that the model’s accuracy is challenged by the relatively large fluctuations observed for some of the simulations; e.g., Sim. No. 1 that had a z-axis rotation of 157.5, cf. Figure 10. This suggests that z-axis rotation positively contributes to the $\bar{\lambda_{MZ}}$ value but also introduces increased uncertainty. Another intriguing finding was the influence of the number of arms; fewer arms resulted in a higher dispersion. As observed in Figure 11 when comparing the average vorticity of Sim. No. 21, 22, and 27, all having identical parameters except for the number of arms, it is evident that the simulations with fewer arms exhibit lower vorticity, which explains the improvement in $\bar{\lambda_{MZ}}$.

#### 3.5.2. Distributive Mixing Index

**Figure 12:**
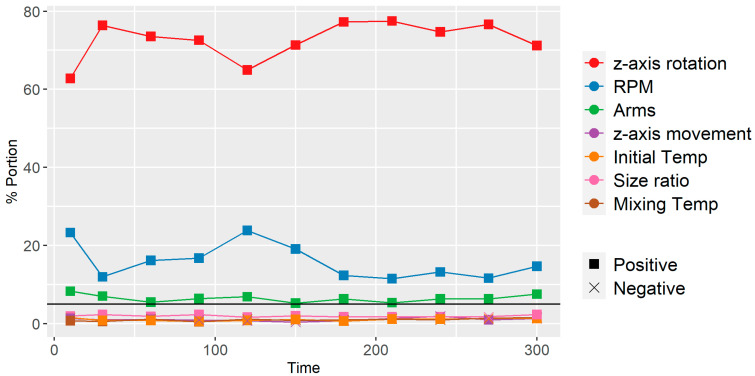
Screening of each parameter to t=10 s and then t=30 s and then every 30 s. Note the significance line at 5%. Positive and negative indicate the way in which the parameter affects the mixing when increased.

**Figure 13:**
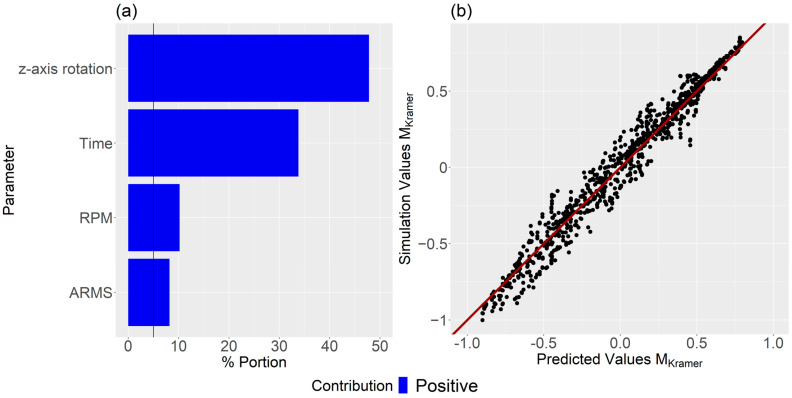
(**a**) Contribution for each parameter, and (**b**) the predicted vs the simulated values. Note the significance line at 5% in (**a**).

In contrast to dispersive mixing, time significantly affected the distributive mixing index, as evidenced in Figure 7. Thus, predictive screenings for $M_{Kramer}$ were conducted at specific time steps across all simulations: first at 10 s, then at 30 s, followed by 30-s intervals. The aim was to identify which parameters, on average, had the most influence on $M_{Kramer}$. As seen from Figure 12, the z-axis rotation is the dominant factor after 10 s and RPM is the second dominating factor. The arms are 5.1%, which is above the significance line. Thus, the bootstrap forest parameters consisted of time, z-axis rotation, RPM, and arms. Figure 13a shows that all dominant parameters contribute positively to mixing when increased. In addition, there is good coherence between the predictive $M_{Kramer}$ values and the simulated $M_{Kramer}$ values, as seen in Figure 13b. The $R^{2}$ for the validation set was 0.949.

There were two interesting observations, first, z-axis rotation is more influential on mixing than time, and second, increasing the number of arms has a positive effect on the distributive mixing index, which is opposite as compared to the dispersion mixing index. This indicates that one needs to select the number of arms carefully in a change can mixer depending on whether the material at hand requires a focus on distributive or dispersive mixing. Additionally, even in small timestep intervals, neither the initial nor the mixing temperature shows to have any influence on the distributive mixing index, further highlighting the paramount importance of the mechanical parameters.

## 4. Conclusions

This study examined the impact of eight parameters on dispersive and distributive mixing in a change can mixer, utilizing both CFD models and the machine learning technique known as the bootstrap forest algorithm. A total of 35 simulations with each 26 time-dependent data points constituted the dataset for this research. The CFD models yielded results in accordance with theoretical expectations concerning fluid velocity and temperature; however, certain simulations revealed elevated average fluid temperatures compared to the set mixing temperatures, which can be attributed to viscous dissipation.

- Four key parameters significantly influenced dispersive mixing, represented by the average value of $\lambda_{MZ},\;\bar{\lambda_{MZ}}$, achieving an $R^{2}$ value of 0.836 in the predictive model. Similarly, distributive mixing, denoted by $M_{Kramer}$, had an $R^{2}$ value of 0.949 in the predictive models.
- The z-axis rotation and RPM positively affected both mixing indexes, with z-axis rotation showing the greatest impact but also increased uncertainty.
- The number of arms negatively influenced dispersive mixing but positively impacted distributive mixing. The size ratio negatively affected dispersive mixing, while time significantly influenced distributive mixing.
- The z-axis movement, mixing temperature, and initial temperature demonstrated no significant effect in this study.

These findings provide essential direction for the refinement of mixing operations in industrial contexts. Future work could explore the validity of these simulations by adjusting the Z-axis rotation within the mixer, as such adjustments are anticipated to enhance both dispersive and distributive mixing. Subsequent research may benefit from validating these results in actual mixing scenarios, as well as assessing their relevance to different mixer types and materials. The investigative approach adopted herein offers a foundational framework for analogous future research in mixing science.

## Figures and Tables

**Table 1:** Physical data for the mixed fluid.

Name of the Property	Values	Unit
Density	1133	$\frac{kg}{m^{3}}$
Heat capacity	1389	$\frac{J}{kg\cdot {}^{\circ}C}$
Heat conductivity	$\begin{array}{c} k\left(T\leq 23\;{}^{\circ}C\right)=0.52\\ k\left(23\;{}^{\circ}C<T<71\;{}^{\circ}C\right)=-1.88\cdot 10^{-3}\;T+0.56\\ k\left(T\geq 71\;{}^{\circ}C\right)=0.43 \end{array}$	$\frac{J}{m\cdot K}$

**Table 2:** Viscosity data of the simulated fluid.

Symbol	Value	Unit
$\tau_{0}$	2	Pa
n	0.415	-
$\mu_{0}$	29,307	Pa·s
a	0.853	-
c	0.00175	°C
$T^{ref}$	82.557	°C
$\gamma_{c}$	0.14	$s^{-1}$

**Table 3:** Numerical DOE.

Sim. No.	Init. Temp	Mix Temp	RPM	Z-Axis Rotation	Z-Axis Movement	Arms	Size Ratio
1	50	80	22.5	157.5	6	3	1
2	50	20	3	0	0	2	0.667
3	80	50	30	0	0	2	1
4	20	50	3	21	6	3	0.667
5	80	20	16.5	0	6	3	1
6	20	80	16.5	115.5	0	2	0.667
7	80	80	30	150	6	2	0.667
8	20	20	3	15	0	3	1
9	80	20	3	21	6	2	0.75
10	20	80	30	0	0	3	0.75
11	80	80	3	0	3	3	0.667
12	20	20	22.5	157.5	3	2	1
13	80	80	3	21	0	2	1
14	20	20	30	0	6	3	0.667
15	80	20	22.5	157.5	0	3	0.667
16	20	80	3	0	6	2	1
17	50	50	16.5	82.5	3	2	0.75
18	50	50	16.5	82.5	3	3	0.75
19	80	20	10	10	0	2	0.75
20	80	20	10	10	0	3	0.75
21	80	20	10	30	0	2	1.1
22	80	20	10	30	0	3	1.1
23	80	20	20	20	0	2	1.1
24	80	20	20	20	0	3	1.1
25	80	20	20	60	0	2	0.75
26	80	20	20	60	0	3	0.75
27	80	20	20	20	0	1	1
28	20	80	16.5	115.5	0	1	1
29	80	20	22.5	157.5	0	1	1
30	80	20	10	30	0	1	1.1
31	80	80	3	0	3	1	0.667
32	50	50	16.5	82.5	3	1	0.75
33	80	20	10	30	0	2	0.75
34	80	20	10	50	0	2	0.75
35	20	20	3	0	0	1	1.1

## Data Availability

The original contributions presented in the study are included in the article, further inquiries can be directed to the corresponding author.
